# Genomic analysis of bovine respiratory disease resistance in preweaned dairy calves diagnosed by a combination of clinical signs and thoracic ultrasonography

**DOI:** 10.1371/journal.pone.0318520

**Published:** 2025-03-21

**Authors:** Maria G. Strillacci, Vincenzo Ferrulli, Francesca Bernini, Davide Pravettoni, Alessandro Bagnato, Ilaria Martucci, Antonio Boccardo

**Affiliations:** Department of Veterinary Medicine and Animal Science, Università degli Studi di Milano, Via dell’Università 6, Lodi, Italy; Michigan State University, UNITED STATES OF AMERICA

## Abstract

Bovine respiratory disease (BRD) poses a significant risk of morbidity and mortality in preweaned dairy calves. Research indicates that this multifactorial disorder can be attributed to the involvement of various pathogens. Currently, there is little information from genome-wide association studies (GWAS) for BRD resistance in young calves based on objective measures and classification of the disease. In this study, we moved forward in phenotyping BRD by coupling two diagnostic tests, the thoracic ultrasonography (TUS) and Wisconsin respiratory score (WISC), in order to assess susceptible and resistant animals to BRD. A total of 240 individuals were scored for BRD using TUS and WISC. A GWAS was performed using a selective genotyping approach to identify Quantitative Trait Loci (QTL) for BRD resistance. A total of 47 calves classified as BRD resistant (TUS ≤  1/ WISC ≤  4) and 47 as BRD susceptible (TUS =  5/ any WISC) were genotyped with the NEOGEN’s GGP Bovine 100K SNP chip. QTL were then identified comparing the SNPs allelic frequencies between the two groups. A total of 28 QTL regions (QTLRs) were defined according to significative SNPs, 141 genes were annotated in the defined QTLRs. The genes were functionally classified into 4 main categories, i.e., i) regulation of systemic arterial blood pressure, ii) fertility, iii) immune function, and iv) filament cytoskeleton. Furthermore, 61 out of 141 genes identified here can be considered promising candidate genes since they were already associated with BRD resistance in published GWAS studies in dairy cattle. The ASB9, BMX, EPSTI1, and OLFM4 genes were identified in 4 of the 6 considered studies. This study paves the way for further research to mine the genome for resistance to respiratory diseases, utilizing an accurate classification process.

## Introduction

Bovine respiratory disease (BRD) is a common medical condition that affects cattle and causes significant economic losses worldwide [1]. It is also one of the main reasons for using antimicrobials in cattle [2]. Despite the scientific community’s extensive focus on BRD in recent decades, preweaned dairy calves’ morbidity and mortality are still estimated at 12% and 14%, respectively, in the United States [3,4]. The occurrence of BRD in calves is likely attributed to several significant factors related to this disease. Bovine respiratory disease is, in fact, a multifactorial disorder with known predisposal factors and environmental components, host-pathogen interaction, and external stressors. The immune system plays an important role in influencing the individual immune response, development, and progression of the disease throughout the individual’s life [5].

A significant challenge in managing BRD is the lack of a field gold-standard diagnostic tool for discriminating affected vs. non affected individuals in preweaning calves and older individuals. Recent studies have compared thoracic ultrasonography (TUS) with clinical signs to enhance the diagnosis of BRD. The findings indicate that TUS is an accurate method for the in vivo assessment of lung lesions during BRD-related suppurative bronchopneumonia episodes, the most common lower respiratory tract infection in young dairy calves. Thoracic ultrasonography TUS has demonstrated good inter-rater agreement [6] and satisfactory accuracy in detecting active bronchopneumonia [7,8]. Longitudinal studies focusing on lung lesions assessed by TUS in preweaned dairy calves have shown that farm management practices, including the types of rearing, affected the dynamics of the morbidity and the severity of the disease [9–12].

To our knowledge, genome-wide association studies (GWAS) for BRD in calves using lung lesions detected by TUS combined with a clinical scoring system was recently studied in US Holstein [13]. Similarly, a recent study reported nineteen differentially expressed genes in peripheral leukocytes of preweaned Holstein calves with respiratory disease diagnosed with TUS and clinical signs [14]. Another GWAS for BRD resistance on objective measures was performed by Lipkin et al. (2018) [15], who recorded lung lesions at the slaughterhouse and identified QTL related to BRD resistance. These findings support that a genetic component is involved in individual immune response, and the estimated heritability for different pathogen susceptibility ranges from 0.04 to 0.24 [13,16–20].

In many studies, the evaluation of the BRD phenotype relies on subjective assessments of clinical signs or drug treatments reported by breeders. However, these assessment methods may not specifically address lower airway disease, the primary pathological challenge associated with BRD. A classification system that combines TUS and clinical signs can help mitigate the limitations of existing field data, mainly when performed in several herds, excluding a confounding effect between genetic and environmental factors [21]. Additionally, GWAS based on a selective genotyping approach allows the creation a case-control design by selecting the two extreme tails of the recorded phenotypes [22]. This experimental design, in association with comparison of allele frequencies of markers in the two groups [23], showed its effectiveness in the discovery of quantitative trait locus (QTL) in many studies [24–26] and was applied previously by Lipkin et al. (2018) specifically on BRD resistance [15].

The objectives of this study were i) to score individuals for BRD in several herds using a combination of TUS scoring system and clinical signs as a viable way to collect reliable phenotypic records of resistant and susceptible female calves in field conditions and ii) to use the obtained information in a selective genotyping GWAS to identify the QTL related to this disease.

## Materials and methods

### Animal sampling, genotyping and ethics statement

For this study, the clinical information of 240 Holstein female calves from 10 commercial dairy farms (minimum and maximum number of preweaning calves per herd was 10 and 43, respectively) located in northern Italy were available from previous studies [27,28]. Farms were selected for a history of cough in preweaned Holstein heifers housed in multiple pens with automatic calf feeders, with no registered antimicrobial or anti-inflammatory treatments in the 15 days before the study. Preweaned calves were randomly subjected to a systematic bilateral TUS using ventral landmarks described by [29]. The assessment was performed on the right side at intercostal space [ICS] 10-1 and the left at ICS 10-2 using a portable ultrasound unit equipped with 7.5 MHz linear transducer (Esaote MyLab Five Vet, Esaote S.p.A., Genova, Italy). Data on lung lesions (type of lesions or depth in cm for consolidation) from each intercostal space were manually transferred into an Excel spreadsheet and simultaneously evaluated. The severity of the disease was examined and assessed by the mass of lung tissue involved according to the methods described by [30]. Thoracic ultrasonography has been, therefore, assigned on a 0-to-5-point scale. Briefly: TUS = 0 indicated normal aerated lung parenchyma (with none or few comet-tail artifacts); TUS = 1 indicated diffuse comet-tail artifacts without consolidation; TUS = 2 consisted of lobular or patchy pneumonia, a pulmonary consolidation of > 1 cm depth among normal aerated lung parenchyma; TUS = 3 consisted of a full-thickness consolidation of one lung lobe; TUS = 4 lobar pneumonia affected two lobes; TUS = 5 lobar pneumonia involved three or more lobes. Cranial and caudal aspects of the cranial lobe were evaluated individually. Each enrolled calf underwent clinical evaluation using the Wisconsin Respiratory Score (WISC), where 0 to 3 points (i.e., 0 =  normal, 3 =  severely abnormal) were assigned for each of the following categories: i) rectal temperature; ii) nasal discharge; iii) ocular discharge; iv) cough; v) ear position. The eye and ear scores were considered collectively, and the higher of the two was used. A total WISC was then assigned to each calf as the sum of all categories, i.e., values ranging from 0 to 12.

To perform the association study with the selective genotyping approach, the TUS and WISC scores (BRD score) were used. Out of the 240 scored individuals, calves with a TUS score of 5 were considered susceptible to BRD (S-BRD), and the ones with a TUS score of 0 or 1 coupled with a total WISC score ≤  4 without cough were classified resistant to BRD (R-BRD). S1 Table lists the scores of the 47 S-BRD and the 47 calves classified R-BRD).

Residual blood samples collected for clinical screening by [27,28] were stored at -80°C and then made available for this study as approved by the ethical committee of the University of Milan (approval number 2/16, February 15, 2016). In addition, the study was approved by the Institutional Animal Care and Use Committee of the University of Milan (approval number 104/2020, January 15, 2020).

DNA from the residual blood of selected calves (47 samples of S-BRD and 47 samples of R-BRD, representing the top and bot 20% of the distribution of the scored animals, made this experimental design classifiable as a case vs control study) was extracted using the Quick-DNA Miniprep Kit of Zymo Research (Zymo Research Corporation). After the DNA quality check (DNA integrity, purity and concentration were verified by standard procedures; i.e., 1% Agarose Gel and NanoQuant Infinite m200 - Tecan), and its dilution to a 50 ng/ul, genotyping was performed with the NEOGEN’s GGP Bovine 100K (GeneSeek®) SNP array by an external laboratory. SNPs coordinates were mapped according to the bovine genome assembly ARS-UCD1.2.

### Statistical analysis

To perform a GWAS for BRD, we used the selective genotyping [22] of animals with high versus low BRD scores (R-BRD and S-BRD animals), i.e., the top and bottom 20% of combined TUS and WISC scoring for BRD. Then, we combined the genotype data from the groups of animals at each extreme (R_BRD vs. S-BRD) and analyzed the genotype frequencies as if we had undertaken a DNA pooling [23].

Genotypes of both selected R-BRD and S-BRD cows were randomly divided into two biological replicates for each group (R1-BRD and R2-BRD, S1-BRD and S2-BRD). The allele frequency at a single marker level was obtained for each replicate using the ‘genotype statistics by marker’ function of Golden Helix’s SVS software (SNP and Variation Suite v8.9 module, Golden Helix Inc. Bozeman, MT, USA). Monomorphic and duplicated markers were filtered out because they were non-informative. Additionally, using an in-house R-script, the SNPs lying in the top 5% of the absolute value of allele difference between replicates (i.e., R1-BRD vs. R2-BRD, S1-BRD vs. S2-BRD) were also excluded from the analysis. After these edits, 68,256 SNPs (on autosomes, BTAs) and 2,656 SNPs (on chromosome X, BTX) were available for the association analyses.

Two separate genome association analyses, the first GWAS using autosomal SNPs and the second one using the polymorphism on chromosome X, were performed using an in-house script written in R as reported in [26]. Briefly, the p-value for each marker was calculated as follows:


$$\text{Ztest}=\frac{\text{Dtest}}{\text{SD}\left(\text{Dnull}\right)}$$

Dtest is the difference of the allele frequencies among tails, and Dnull is the difference of the allele frequencies within tails. The GWAS results were visualized through a Manhattan plot. The Bonferroni genome-wide correction was used to set a 5% significance threshold.

### Gene annotation

All SNPs over the 5% Bonferroni threshold were annotated, and the SNP’s rsID code (Reference SNP cluster ID) of each the Illumina SNP marker has been obtained using the multi-species SNPchiMp v.3 database [31]. The Variant Effect Predictor (VEP) tool of the Ensembl database [32] allowed to annotate the significant SNPs through the rsID codes according to the *Bos taurus* genome assembly ARS-UCD1.2 (Annotation Release: 106).

QTL regions (QTLRs) are here defined as the genome centered over single significant SNPs or around more consecutive ones (from 2 to 5 SNPs in our study). When the distances of the consecutive SNPs mapped on the same chromosome were less than 1 Mbp, the gap between the positions of the first and last SNPs was calculated, and the difference respect to 1 Mbp was equally accounted in upstream (from the first SNP) and downstream (from the last SNP) positions, to cover the total length of 1 Mbp (these new positions defined the QTLR boundaries).

The list of genes annotated within these defined QTLRs was downloaded from the NCBI database using the tool integrated into the Genome Data Viewer (https://www.ncbi.nlm.nih.gov/gdv?org=bos-taurus). The functional enrichment analysis was then conducted using the ClueGo and Genemania tools of Cytoscape software [33–35].

### Inbreeding coefficients

In order to investigate possible role of inbreeding on the calf’s capability to cope with an infection disease, for each animal, two different inbreeding coefficients (F_HOM_, based on the excess in the observed number of homozygous genotypes [36] and F_ROH_, based on the runs of homozygosity (ROH), that are continuous genomic homozygous segments inherited from common ancestors [37]) were calculated using the SVS software. ROHs were identified setting as parameters: i) a minimum number of 30 SNPs/ROH; (ii) a minimum ROH length of 1Mb, to avoid the identification of short and common ROH due to Linkage Disequilibrium; (iii) no missing SNPs and no heterozygous genotypes presence in ROH definition. F_ROH_ for all samples were calculated as:


$$F_{ROH}=\frac{\sum_{i}^{n}L_{ROH}}{L_{AUT}}$$

where L_ROH_ is the length of the ith ROH segment, n is the number of detected ROH and L_AUT_ is the length of the autosomal genome covered by the SNPs used in the GWAS.

## Results

The values of WISC and TUS, together with age and farm number of the S-BRD and R-BRD are included in S1 Table.

**Fig. 1:**
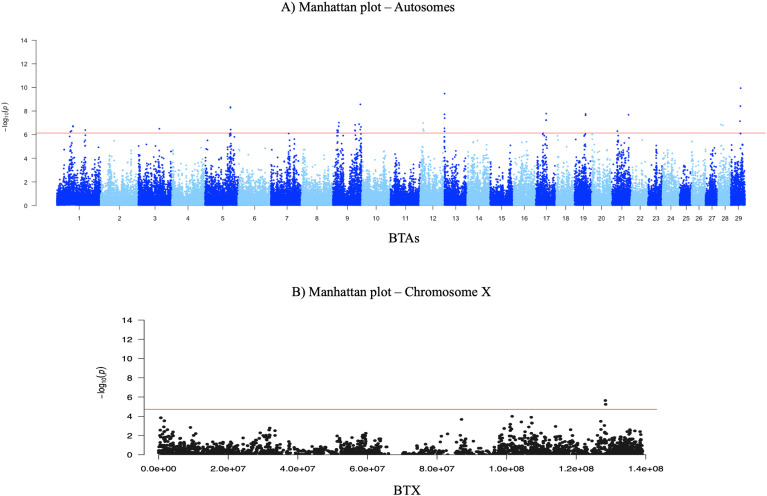
Manhattan plots of the GWAS performed on BTAs (A) and on BTX (B). The Red lines represent the Bonferroni p-value threshold of significance, set at 5% genome wide.

Fig 1 shows the two Manhattan plots of the genome wide association analyses performed on BTAs (A) and BTX (B) with the Bonferroni (red line) threshold set at 0.05.

Twenty-seven QTLRs, defined by 45 significant SNPs, were identified on 11 autosomes, and only one QTLRs was defined on BTX by 2 significant SNPs, as reported in Table 1. For nineteen SNPs the p-value was greater than 1.40E-07, as being located over the Bonferroni multiple testing correction threshold of 1%. The highest number of QTLRs was identified on BTA 9 (n. 8). The significant markers were annotated as intronic markers (n. 21), 3_prime_UTR variant (n. 1), and intergenic SNPs (n. 21).

**Table 1:** QTLRs identified in this study. Start and End position of the QTLRs are in concordance with the ARS-UCD1.2 assembly.

QTLR_n.	Start	End	SNP	RS SNP code	CHR	BP	distance	P value	Gene	SNP Position	upstream	in-between	downstream
QTLR_1	48950364	49950364	BovineHD0100014033	rs3423105562	1	49450364		5.69E-07		intergenic			ALCAM
QTLR_2	52682936	53682936	BovineHD0100015084	rs3423105354	1	53182936	3732572	4.80E-07	MYH15	intron	IFT57, HHLA2		DZIP3, CIP2A, TRAT1
QTLR_3	58199902	59199902	Hapmap47450-BTA-96948	rs41592061	1	58626471	5443535	2.02E-07	ZDHHC23	intron	SIDT1, USF3, NAA50, ATP6V1A, GRAMD1C		DRD3, TIGIT, MIR568
			BovineHD0100016729	rs109974499	1	58678558	52087	1.85E-07	CCDC191	intron			
			BTA-96949-no-rs	rs41663660	1	58773333	94775	2.02E-07	QTRT2	intron			
QTLR_4	102359117	103359117	BTA-39406-no-rs	rs3423093242	1	102859117	44085784	4.05E-07		intergenic	SI		
QTLR_5	75188352	76188352	BovineHD0300022027	rs109698273	3	75688352		3.13E-07		intergenic	LRRC7		
QTLR_6	91226361	92226361	BovineHD0500026165	rs5369491638	5	91695368		4.68E-09	PIK3C2G	intronic	CAPZA3, PLCZ1, PIK3C2G	PIK3C2G	PIK3C2G, RERGL
			UA-IFASA-4080	rs29027239	5	91757353	61985	5.16E-09	PIK3C2G	intronic			
QTLR_7	92296641	93296641	ARS-BFGL-NGS-116999	rs110267314	5	92796641	1039288	3.71E-07		intergenic			LMO3
QTLR_8	15128799	16128799	ARS-BFGL-NGS-1357	rs109782091	9	15129365		4.22E-07	FILIP1	intron	FILIP1	SENP6, MYO6, IMPG1	
			BovineHD0900004394	rs133351462	9	16128232	998867	6.46E-07		intergenic			
QTLR_9	17082837	18082837	BovineHD0900004814	rs135595961	9	17544244	1416012	4.22E-07	MEI4	intron	HTR1B, MEI4		
			BovineHD0900004841	rs136778805	9	17621429	77185	4.41E-07		intergenic			
QTLR_10	18370767	19370767	ARS-BFGL-NGS-46104	rs110957535	9	18870767	1249338	5.94E-07		intergenic	IRAK1 BP1, PHIP, HMGN3		LCA5, SH3BGRL2
QTLR_11	20739564	21739564	BovineHD0900005695	rs42252864	9	20825772	1955005	1.94E-07		intergenic			FAM46A
			BTB-00384566	rs43588529	9	21653355	827583	9.50E-08		intergenic			
QTLR_12	79422330	80422330	BovineHD0900022313	rs137663324	9	79439745	57786390	4.22E-07		intergenic		NMBR, GJE1, VTA1, ADGRG6, MIR2284AA-4,HIVEP2	
			BovineHD0900022633	rs109987604	9	80355614	915869	4.60E-07	HIVEP2	intron			
			BovineHD0900022635	rs135739881	9	80357230	1616	4.60E-07	HIVEP2	intron			
			BovineHD0900022654	rs43732735	9	80404914	47684	1.49E-07	HIVEP2	intron			
QTLR_13	94091694	95091694	ARS-BFGL-NGS-103694	rs109848700	9	94591694	14186780	1.30E-07	SYNJ2	intron	ZDHHC14, SNX9, SYNJ2		SERAC1U60, GTF2H5, TULP4, TMEM181, DYNLT1, SYTL3
QTLR_14	98308661	99308661	BovineHD0900028984	rs110848300	9	98567810	3976116	3.71E-07		intergenic		QKI	
			BovineHD0900029149	rs109299906	9	99049511	481701	2.72E-09		intergenic			
QTLR_15	100708383	101708383	BovineHD0900030047	rs109315371	9	101208383	2158872	2.21E-07		intergenic	PDE10A, TBXT		PRR18, SFT2D1, MPC1, RPS6KA2
QTLR_16	10342117	11342117	BovineHD1200003098	rs110241533	12	10835926		3.41E-07		intergenic	OLFM4		PCDH8, MIR759, CNMD, SUGT1, ELF1, WBP4, KBTBD6
			BovineHD1200003101	rs109988176	12	10848307	12381	1.04E-07		intergenic			
QTLR_17	12562194	13562194	BovineHD1200003802	rs109433223	12	13062194	2213887	4.80E-07	EPSTI1	intron	TNFSF11, FAM216B		DNAJC15, ENOX1
QTLR_18	824812	1824812	BovineHD1300000152	rs137599388	13	1033957		2.87E-07	PLCB1	intron	PLCB1	MIR2285M-1	PLCB1
			BovineHD1300000159	rs136868008	13	1055763	21806	1.89E-08	PLCB1	intron			
			BovineHD1300000219	rs134875747	13	1205835	150072	5.23E-07	PLCB1	intron			
			BovineHD1300000319	rs135872126	13	1526992	321157	4.00E-08	PLCB1	intron			
			BovineHD1300000354	rs109072042	13	1615667	88675	3.33E-10	PLCB1	intron			
QTLR_19	36558263	37558263	BovineHD1700010310	rs110869101	17	36952264		6.04E-08	FSTL5	intron	FSTL5	FSTL5	FSTL5
			BovineHD1700010314	rs109399341	17	36959999	7735	5.77E-08	FSTL5	intron			
			Hapmap43738-BTA-99532	rs41567083	17	37164261	204262	1.64E-08	FSTL5	intron			
QTLR_20	40272628	41272628	BovineHD1900011769	rs137486957	19	40711083		2.28E-08	TNS4	intron	LRRC3, GSDMA, PSMD3, CSF3, MED24, THRA, NR1D1, MSL1, CASC3, RAPGEFL1, WIPF2, CDC6, RARA, GJD3, TOP2A, IGFBP4	CCR7	KRT22, KRT24, KRT25, KRT26, KRT27, KRT28, KRT10, KRT12, KRT20, KRT23, KRT39, KRT40, KRTAP3-3, KRTAP3-1, KRTAP1-1
			Hapmap54523-ss46526232	rs41255371	19	40834172	123089	1.75E-08	SMARCE1	3_prime_UTR_variant			
QTLR_21	19594619	20594619	BovineHD2100005997	rs137755071	21	20094619		5.01E-07		intergenic	AEN, ISG20		ACAN, HAPLN3, MFGE8, ABHD2
QTLR_22	59783958	60783958	BovineHD2100018046	rs110097968	21	60283958	40189339	2.08E-08	SYNE3	intron	DICER1, CLMN		GLRX5, TCL1B, TCL1A
QTLR_23	9684831	10684831	BovineHD2800003135	rs135642552	28	10184831		1.42E-07	RYR2	intron	RYR2		RYR2, ZP4
QTLR_24	16953971	17953971	BovineHD2800004904	rs135022518	28	17453971	7269140	1.62E-07		intergenic	TMEM26		CABCOCO1, ARID5B
QTLR_25	32764941	33764941	BovineHD2900010039	rs3423598291	29	33264941		7.25E-08		intergenic	JAM3, IGSF9B, SPATA19		OPCML
QTLR_26	34222160	35222160	BovineHD2900010637	rs42183295	29	34722160	1457219	3.84E-09	NTM	intron	OPCML		NTM
QTLR_27	35244229	36244229	BovineHD4100019037	rs136936924	29	35744229	1022069	1.15E-10		intergenic	NTM		
QTLR_28	127916138	128916138	BovineHD3000038779	rs109808194	X	128374647		2.31E-06		intergenic	ACE2, BMX, PIR, VEGFD, PIGA, ASB11, ASB9		MOSPD2, FANCB, GLRA2
			BovineHD3000038809	rs134798226	X	128457628	82981	5.77E-06		intergenic			

QTLR, serial number of the QTLR; Length, length of the QTLR in bp; Distance, distance between the first and the last SNP of a QTLR located on the same chromosome; Upstream, In-between, Downstream, are genes mapped within the QTLR.

A total of 141 annotated genes (protein-coding genes) were found, including 4 miRNA (Table 1). Furthermore, 53 and 59 genes were distributed respectively in upstream and downstream positions respect to significant SNPs, 20 genes were the ones in which significant SNPs mapped, and 9 genes covered all (e.g., PLCB1 and FSTL5) or part (e.g., PIK3C2G, FILIP1, MEI4, SYNJ2, RYR2, OPCML, and NTM) of the QTLR_1 Mbp length region.

**Fig. 2:**
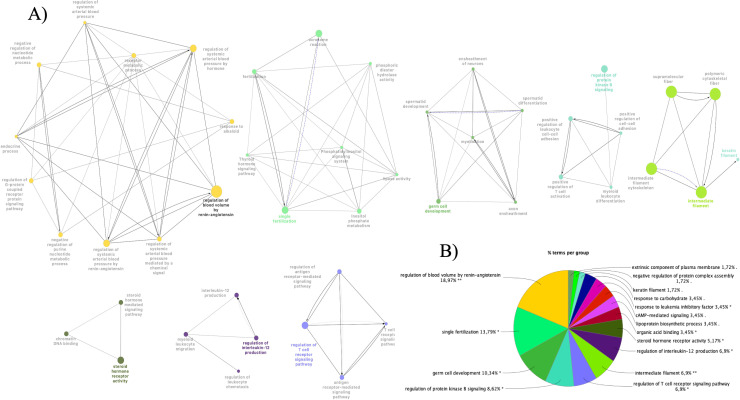
Annotation Results. ClueGo Gene networks with at least three terms (A); Go-terms with higher % of associated genes (B) (Complete list of functional terms is reported in S2 Table).

Out of the 141 genes, 62 were present and annotated with Cytoscape software and subsequently included in the gene networks shown in Fig 2 (only networks with at least 3 terms were displayed). All these genes were significantly (P ≤ 0.05) belonging to 58 GO Terms (n. 55) and KEGG pathways (n. 3), and 57 (92%) appeared in more than one term (S2 Table), mainly involved in immune response, fertility and blood pressure regulation processes.

### Inbreeding coefficients

The descriptive statistics for F_HOM_ and F_ROH_ coefficients are reported in Table 2. Both coefficients resulted slightly lower in R-BRD than those calculated for S-BRD calves.

**Table 2:** Inbreeding coefficients calculated for R-BRD and R-BRD calves.

Group	F_HOM_	F_HOM_	F_ROH_	F_ROH_
	Mean (SD)	Min–Max values	Mean (SD)	Min–Max values
R-BRD	-0.013 (0.037)	-0.083–0.094	0.155 (0.031)	0.097–0.233
S-BRD	0.008 (0.056)	-0.078–0.301	0.170 (0.045)	0.093–0.401

## Discussion

The key factor in disclosing QTL associated with resistance or susceptibility to a specific disease is the correct classification based on a chosen phenotype. In our study, calves were identified as affected or non-affected by BRD using a quantitative scoring system derived from the combination of TUS, a noninvasive method for the visualization of lung lesions with high diagnostic accuracy [38,39], and clinical signs scored with WISC. The TUS scoring method is recognized to have a high sensitivity and specificity [9,40] and allows the classification of calves in all 5 TUS scoring across the herds. It is worth mentioning that the classification obtained for BRD resistance is objective and can be proposed as a field classification method to score preweaning calves.

We assumed that the R-BRD and S-BRD calves underwent the same management factors and environmental conditions: within each herd, they were farmed in the same pens, closely located, and, therefore, assumed to be equally exposed to BRD pathogens. It is also to be noticed that they were similar in age and had at least 15 days of minimum exposure to pathogens in the same pen.

Additionally, the 10 herds here used were sampled among a much wider number of farms routinely visited by the Veterinary Mobile Clinic of our Department.

As calves were scored between February 2021 and February 2022, we can identify individuals still in production after three years among those genotyped. The survival rate of calves (now heifers or first calving cows) was verified by consulting the ANAFIBJ website (Associazione Nazionale Allevatori della Razza Frisona, Bruna e Jersey Italiana), which allows access to information on the animals genotyped and registered in the herd book using the identification number. The survival rate of female calves was notably higher for R-BRD calves (80%) compared to S-BRD ones (56%). While this survival rate is not directly linked to BRD, it does raise the possibility of BRD’s influence on the female population in the herd. The literature reports among the risk factors involved in BRD mortality the inbreeding level, possibly related to inbreeding depression (ID) [41,42]. We have calculated inbreeding based on genomic information, i.e., F_HOM_ and F_ROH_. Even if both coefficients resulted slightly lower in R-BRD calves, the differences in F_HOM_ and F_ROH_ cannot explain by per se the diverse capability of an animal to cope with this multi pathogen disease.

In this study, 47 significant SNPs were associated with BRD susceptibility vs. resistance. The most significant SNP, the rs109072042, was found in the QTLR_18 where other 5 significant SNPs mapped in intronic positions of the PLCB1 gene on BTA13. This gene seems to play a role in inhibiting endothelial inflammation, and as reported by [43] the expression of PLCB1 is positively correlated with chronic obstructive pulmonary disease. On BTA9, more significant SNPs (n. 15) were mapped, and three were located within the HIVEP2 gene. Schumann et al. (2020) reported that this gene influenced regulatory T cell function in suppressing inflammation [44]. On the same BTA some significant SNPs were annotated in intronic position, one in the FILIP1 gene, involved in skeletal muscle cell differentiation [45], three in the FSTL5 gene, implicated in activation specific protein of the caspase pathway mediating the apoptosis process [46], and one in the EPSTI1 gene, a modulator of macrophage activation with a suggested role in immunotherapies against inflammatory diseases [47]. The functional gene annotation, conducted with the ClueGO tool, revealed that the candidate genes annotated in the 28 QTLRs can be grouped into four main categories: regulation of systemic arterial blood pressure, fertility, immune function, and filament cytoskeleton. These categories and their associated genes could play a significant role in determining susceptibility vs. resistance to BRD. The ACE2 gene, assigned to the regulation of systemic arterial blood pressure (S2 Table), is well-known to be involved in different respiratory diseases such as COVID-19, severe acute respiratory syndrome (SARS), and Influenza [48], having an important role in body homeostasis, not only through the blood pressure regulation but also with the electrolyte balance [49]. Nine of the 13 genes assigned to immune function were already associated with BRD, as reported in S2 Table. Among the other four genes, TIGIT and CSF3 could affect the efficiency of the immune response against BRD pathogens with an opposite contribution. In fact, CSF3 gene encodes for G-CSF growth factor that regulates different aspects of neutrophil biology. A study in mice reported that a loss of G-CSF signaling impaired neutrophil-mediated immunity and caused an increased susceptibility to challenge bacterial pathogens [50]. TIGIT seem to promote T cell dysfunction by altering their phenotype and cytokine profile during chronic viral infection [51].

**Table 3:** List of genes annotated in QTLRs (Table 1) already associated with BRD in previous studies.

QTLR_N	Genes	Tizioto et al. 2015 [52]	Lipkin et al. 2017 [15] and 2024 ^*^	Johnston et al. 2019 [53]	Scott et al. 2021[54]	Li et al. 2022 [55]	Green et al. 2023 [56]
QTLR_1	ALCAM	BVDV, IBR					AUCvsDIR
QTLR_2	HHLA2						AUCvsDIR
QTLR_2	MYH15						AUCvsDIR
QTLR_2	DZIP3	BVDV					
QTLR_2	TRAT1	MYCO, PASTE, MANNHE, IBR					AUCvsDIR
QTLR_3	SIDT1	BVDV, PASTE, IBR					
QTLR_3	NAA50	BVDV					
QTLR_3	GRAMD1C						AUCvsDIR
QTLR_3	ZDHHC23	BVDV, IBR					
QTLR_3	CCDC191						AUCvsDIR
QTLR_8	FILIP1	BVDV, MANNHE					
QTLR_8	MYO6	BVDV, MANNHE, IBR					
QTLR_10	IRAK1BP1	BVDV, MANNHE					
QTLR_10	PHIP	BVDV, PASTE, IBR					
QTLR_10	HMGN3			BRSV vs CT			
QTLR_11	FAM46A						AUCvsDIR
QTLR_12	VTA1						AUCvsDIR
QTLR_12	HIVEP2	BVDV, IBR					
QTLR_13	TULP4	IBR					
QTLR_13	DYNLT1	MANNHE, IBR					
QTLR_13	SYTL3	IBR					
QTLR_14	QKI	BVDV, IBR					
QTLR_15	PDE10A	IBR					
QTLR_15	RPS6KA2		RPS6KA2				
QTLR_16	OLFM4	PASTE, MANNHE		BRSV vs CT		BRD vs NO-BRD	AUCvsDIR
QTLR_16	PCDH8						AUCvsDIR
QTLR_16	CNMD						AUCvsDIR
QTLR_16	ELF1	BVDV					
QTLR_16	KBTBD6	BVDV, IBR					
QTLR_17	FAM216B			BRSV vs CT			AUCvsDIR
QTLR_17	EPSTI1	MANNHE, IBR			IBR		AUCvsDIR
QTLR_17	DNAJC15						AUCvsDIR
QTLR_17	ENOX1						AUCvsDIR
QTLR_18	PLCB1	MANNHE					AUCvsDIR
QTLR_20	GSDMA	BVDV, MANNHE, IBR					
QTLR_20	NR1D1	BVDV					
QTLR_20	MSL1						AUCvsDIR
QTLR_20	CASC3				BRSV		
QTLR_20	WIPF2	BVDV, IBR					
QTLR_20	CDC6	MANNHE		BRSV vs CT			
QTLR_20	RARA						AUCvsDIR
QTLR_20	TOP2A	BVDV, IBR			IBR, MYCO, MANNHE		
QTLR_20	IGFBP4	BVDV, PASTE, MANNHE, IBR					
QTLR_20	CCR7	BVDV, MANNHE					
QTLR_20	KRT23						AUCvsDIR
QTLR_21	ISG20	MANNHE, IBR					AUCvsDIR
QTLR_21	MFGE8	MYCO, MANNHE, IBR					
QTLR_21	ABHD2						AUCvsDIR
QTLR_22	DICER1	BVDV, IBR					
QTLR_22	CLMN						AUCvsDIR
QTLR_24	ARID5B	BVDV, IBR					
QTLR_25	JAM3	BVDV, MYCO, PASTE, MANNHE					AUCvsDIR
QTLR_28	ACE2	BVDV, PASTE, MANNHE	ACE2				
QTLR_28	BMX	BVDV, PASTE, MANNHE	BMX			BRD vs NO-BRD	
QTLR_28	PIR	BVDV	PIR				
QTLR_28	VEGFD		VEGFD				
QTLR_28	PIGA	IBR	PIGA				
QTLR_28	ASB11		ASB11				
QTLR_28	ASB9	BVDV, MYCO	ASB9	BRSV vs CT			
QTLR_28	MOSPD2	IBR					
QTLR_28	FANCB	BVDV, PASTE, IBR					

Abbreviations: IBR: bovine rhinotracheitis; BVDV: bovine viral diarrhea virus; BRSV: bovine respiratory syncytial virus; MANNH: Mannheimia haemolytica; PASTE: Pasteurella multocida; MYCO: Mycoplasma bovis; CT: control animal; AUC vs DIR: cattle which experienced a commercial auction setting (AUC) vs cattle from the cow–calf phase (DIR).

*Paper submitted and under revision: Study performed on BRD using SNPs mapped on BTX.

Both in humans and cattle, the interplay between immunology and fertility has been reported in several studies, and it is well-known that reproductive hormones are important for the immune system function [57], as well as that the immune system affects fertility efficiency and subsequently the pregnancy outcomes [58]. For instance, the ABHD2 gene is considered a modulator of sperm fertility and it is also involved in virus propagation and immune response [59]. Different functions have also been recognized for the MFGE8 gene, including promoting the apoptotic cells’ phagocytosis and angiogenesis and its implication in human implantation as well as in other species [60,61]. Finally, the Keratin protein genes (KRTs, n. 15) annotated in the downstream position of QTLR_20 and added within the filament cytoskeleton category by ClueGO are abundant in lung epithelial cells and act modulating the cell functions as receptor signaling, protein proliferation and migration and, inflammatory and immune responses [62].

The comparison with the current literature (Table 3), focused on discovering the genetic basis of BRD resistance or susceptibility, allowed us to identify 61 protein-coding genes (45% of the 141 genes annotated within our QTLRs) that were already associated with this trait. Among these genes, 14 were found in at least two different studies, and ASB9, BMX, EPSTI1, and OLFM4 were reported as common candidate genes in three and four studies focused on BRD, respectively. The BMX gene also resulted as one of the differentially expressed genes identified when comparing BRD with non-BRD animals, as reported by [63]. Interestingly, the largest number of genes found in this study and literature refers to Tizioto et al. (2015), who identified genes involved in immune response after an experimental challenge with BRD agents, making this approach a possible benchmark for a case control study for BRD [52]. We may speculate that the phenotyping strategy used here to cluster S-BRD vs. R-BRD was accurate because our results largely overlap those by Tizioto et al. (2015) found in experimental conditions [52].

The success in GWAS studies to identify QTL related to phenotype variation is in fact directly related to the capability to objectively score a trait. This is very simple for quantitative measures such as milk yield, but much more difficult for complex traits related to immune response. Among these traits, BRD or other disease related traits have great impact in dairy cattle, where knowledge of genomic component may open the opportunities and challenges in sustainability of farming systems [21].

**Fig. 3:**
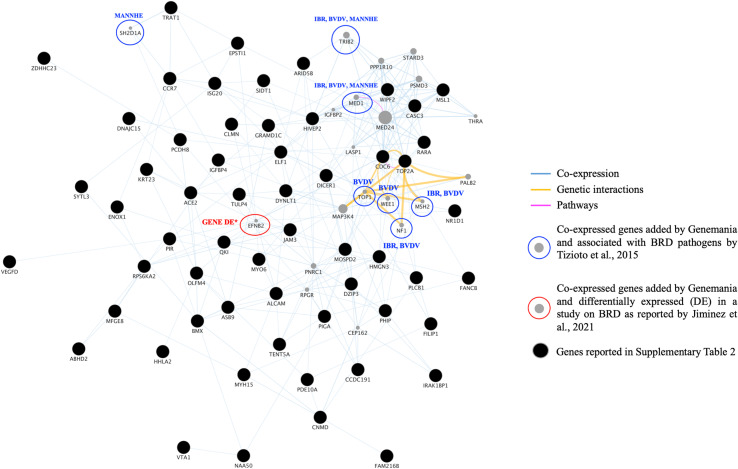
Genes interacting found by Genemania tool implemented in Cytoscape. Co-expressed genes are the ones found in the entire Cytoscape database (*Homo sapiens* species as background).

Fig 3 shows a gene network built with the Genemania tool (implemented in Cytoscape) using 58 of the 61 genes already associated with BRD (the ones reported in Table 3, black full circle in Fig 3). In the same network, the gray full-circle genes (n. 20) were added by Genemania tool representing the potential interaction partners predicted genes according to the references available in its database (background *Homo sapiens*). Seven of these gray genes (SH2D1A, TRIB2, MED1, TOP1, WEE1, MSH2, and NF1) resulted differentially expressed comparing the control and various-pathogen-challenged animals involved in BRD [52], along with the EFNB2 (gray gene circled in red) resulted differentially expressed in a different study performed with healthy and BRD affected animals [63], supporting our results in identifying QTL involved in immune response to BRD.

This investigation utilized a one-gate reverse flow design across all enrolled farms, ensuring that both cases and controls were sampled from a uniform source population with comparable exposure to BRD-related pathogens. This approach yielded unbiased results that may be generalizable to a broader context [64]. However, the observational nature of the study limits our understanding of the temporal development of lung lesions within the sample, thereby restricting our interpretation to the innate immune capacity (i.e., excluding adaptive immunity) to cope with BRD. A longitudinal assessment of these lesions through TUS could have provided additional insights, such as whether a cow remained resistant to BRD or became susceptible over time. Therefore, further research collecting longitudinal data on BRD under similar environmental conditions may validate the findings of this study while also addressing potential adaptive immune resistance to BRD.

## Conclusions

In this study, 240 Holstein calves were scored with TUS and WISC to diagnose BRD. These tools allowed us to identify resistant vs. susceptible calves objectively. Phenotyping of disease traits is one of the critical steps in determining the genomic basis of the immune response to pathogens. In this study, the GWAS results are largely overlap the ones of a study developed in controlled experimental conditions to identify genes related to immune response to BRD, indicating that the clustering based on the score attributed well match the controlled conditions. As the exploitation of genomes through association with traits is a fundamental pillar of the correct phenotype assessment, we may relate the comprehensive list of genes identified in other research to the accuracy of the classification method used here. We may also speculate that the TUS scoring is a valid option for classifying animals across studies, recalling that it is based on an objective classification.

The Cytoscape software used 141 genes in total, and 40% of them appeared in more than one GO term involving the immune response, fertility, and blood pressure regulation processes, plus the filament cytoskeleton one, and all these categories and genes may have a role in the resistance or susceptibility to this disease.

The results of this study show promising outcomes, indicating that using TUS diagnosis could be a valuable phenotype for GWAS efforts to understand the genetic variation contributing to BRD. The straightforward TUS approach, combined with WISC scores in field conditions, could become a standard method for phenotyping calves in herds. Genotyping all individuals with SNP chips could offer additional information and potential longitudinal data throughout the productive lives of calves and cows, helping to explore further the relationship between innate and adaptive immune responses to BRD.

## Supporting information

S1 Table List of S-BRD and R-BRD calves. (DOCX)

S2 Table Gene functional annotation from David database. (DOCX)
