# Supplementary figures and images for: Genomic analysis of bovine respiratory disease resistance in preweaned dairy calves diagnosed by a combination of clinical signs and thoracic ultrasonography
Source: PLoS One. 2025 Mar 21;20(3):e0318520. doi: 10.1371/journal.pone.0318520 (PMC11927911; doi:10.1371/journal.pone.0318520)

**S1 Table.** List of S-BRD and R-BRD calves.


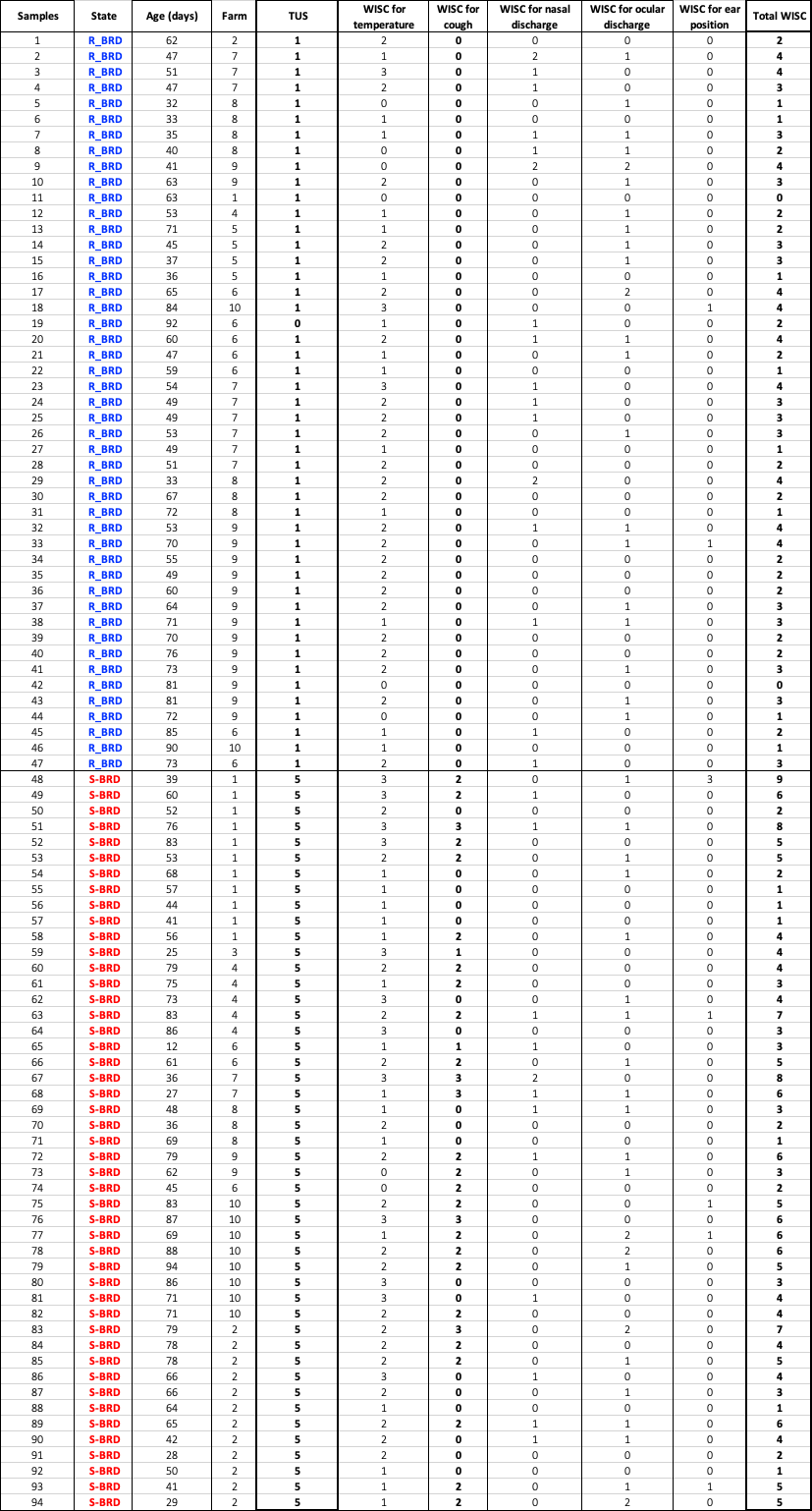

Supplement: S1 Table — (DOCX) [file pone.0318520.s001.docx]
