# Supplementary material for: Genomic analysis of bovine respiratory disease resistance in preweaned dairy calves diagnosed by a combination of clinical signs and thoracic ultrasonography
Source: PLoS One. 2025 Mar 21;20(3):e0318520. doi: 10.1371/journal.pone.0318520 (PMC11927911; doi:10.1371/journal.pone.0318520)
Supplement: S2 Table — (DOCX) [file pone.0318520.s002.docx]

**S2 Table**. Genes functional results provided by Cytoscape Software.

| **ID** | **Term** | **Ontology Source** | **Term PValue** | **Group PValue Bonferroni Corrected (< 0.05)** | **Associated Genes Found** |
| --- | --- | --- | --- | --- | --- |
| GO:0002016 | regulation of blood volume by renin-angiotensin | GO BP | 3.23E-06 | 1.96E-03 | [ACE2, DRD3, RPS6KA2] |
| GO:0003081 | regulation of systemic arterial blood pressure by renin-angiotensin | GO BP | 2.07E-04 | 1.96E-03 | [ACE2, DRD3, RPS6KA2] |
| GO:0001990 | regulation of systemic arterial blood pressure by hormone | GO BP | 9.41E-04 | 1.96E-03 | [ACE2, DRD3, RPS6KA2] |
| GO:0003044 | regulation of systemic arterial blood pressure mediated by a chemical signal | GO BP | 1.97E-03 | 1.96E-03 | [ACE2, DRD3, RPS6KA2] |
| GO:0043112 | receptor metabolic process | GO BP | 1.92E-03 | 1.96E-03 | [ACE2, DRD3, HTR1B, NR1D1, TRAT1] |
| GO:0008277 | regulation of G-protein coupled receptor protein signaling pathway | GO BP | 2.12E-03 | 1.96E-03 | [DRD3, DYNLT1, HTR1B, PLCB1] |
| GO:0043279 | response to alkaloid | GO BP | 2.30E-03 | 1.96E-03 | [DRD3, HTR1B, RYR2] |
| GO:1900543 | negative regulation of purine nucleotide metabolic process | GO BP | 2.87E-03 | 1.96E-03 | [DNAJC15, DRD3, HTR1B] |
| GO:0045980 | negative regulation of nucleotide metabolic process | GO BP | 3.07E-03 | 1.96E-03 | [DNAJC15, DRD3, HTR1B] |
| GO:0003073 | regulation of systemic arterial blood pressure | GO BP | 1.04E-02 | 1.96E-03 | [ACE2, DRD3, RPS6KA2] |
| GO:0050886 | endocrine process | GO BP | 8.76E-03 | 1.96E-03 | [ACE2, DRD3, RPS6KA2] |
| GO:0043401 | steroid hormone mediated signaling pathway | GO BP | 3.12E-03 | 1.18E-02 | [ABHD2, LMO3, NR1D1, RARA, THRA] |
| GO:0007338 | single fertilization | GO BP | 6.02E-04 | 1.61E-02 | [ABHD2, MFGE8, PLCB1, PLCZ1, ZP4] |
| GO:0007340 | acrosome reaction | GO BP | 1.15E-03 | 1.61E-02 | [ABHD2, PLCB1, ZP4] |
| GO:0009566 | fertilization | GO BP | 2.43E-03 | 1.61E-02 | [ABHD2, MFGE8, PLCB1, PLCZ1, ZP4] |
| GO:0032655 | regulation of interleukin-12 production | GO BP | 2.13E-03 | 2.28E-02 | [CCR7, PLCB1, TIGIT] |
| GO:0032615 | interleukin-12 production | GO BP | 2.48E-03 | 2.28E-02 | [CCR7, PLCB1, TIGIT] |
| GO:0097529 | myeloid leukocyte migration | GO BP | 2.67E-03 | 2.28E-02 | [CCR7, JAM3, PLCB1, TNFSF11, VEGFD] |
| GO:0002688 | regulation of leukocyte chemotaxis | GO BP | 2.47E-02 | 2.28E-02 | [CCR7, JAM3, VEGFD] |
| GO:0050856 | regulation of T cell receptor signaling pathway | GO BP | 7.54E-04 | 2.92E-02 | [CCR7, ELF1, TRAT1] |
| GO:0050851 | antigen receptor-mediated signaling pathway | GO BP | 2.92E-02 | 2.92E-02 | [CCR7, ELF1, TRAT1] |
| GO:0050854 | regulation of antigen receptor-mediated signaling pathway | GO BP | 2.13E-03 | 2.92E-02 | [CCR7, ELF1, TRAT1] |
| GO:0050852 | T cell receptor signaling pathway | GO BP | 8.37E-03 | 2.92E-02 | [CCR7, ELF1, TRAT1] |
| GO:0007281 | germ cell development | GO BP | 2.47E-03 | 3.15E-02 | [ABHD2, CAPZA3, JAM3, QKI, RARA, RPS6KA2] |
| GO:0007272 | ensheathment of neurons | GO BP | 2.00E-02 | 3.15E-02 | [ADGRG6, JAM3, QKI] |
| GO:0008366 | axon ensheathment | GO BP | 2.00E-02 | 3.15E-02 | [ADGRG6, JAM3, QKI] |
| GO:0042552 | myelination | GO BP | 1.88E-02 | 3.15E-02 | [ADGRG6, JAM3, QKI] |
| GO:0007286 | spermatid development | GO BP | 9.50E-03 | 3.15E-02 | [ABHD2, CAPZA3, JAM3, QKI] |
| GO:0048515 | spermatid differentiation | GO BP | 1.03E-02 | 3.15E-02 | [ABHD2, CAPZA3, JAM3, QKI] |
| GO:0051896 | regulation of protein kinase B signaling | GO BP | 3.00E-02 | 3.91E-02 | [CSF3, DRD3, TNFSF11] |
| GO:0050870 | positive regulation of T cell activation | GO BP | 2.43E-03 | 3.91E-02 | [CCR7, HHLA2, RARA, TNFSF11, ZP4] |
| GO:1903039 | positive regulation of leukocyte cell-cell adhesion | GO BP | 2.85E-03 | 3.91E-02 | [CCR7, HHLA2, RARA, TNFSF11, ZP4] |
| GO:0002573 | myeloid leukocyte differentiation | GO BP | 4.29E-03 | 3.91E-02 | [CCR7, CSF3, PIR, RARA, TNFSF11] |
| GO:0022409 | positive regulation of cell-cell adhesion | GO BP | 5.60E-03 | 3.91E-02 | [CCR7, HHLA2, RARA, TNFSF11, ZP4] |
| GO:1990823 | response to leukemia inhibitory factor | GO BP | 4.49E-03 | 4.04E-02 | [ARID5B, MPC1, PIGA, TNFSF11] |
| GO:1990830 | cellular response to leukemia inhibitory factor | GO BP | 4.49E-03 | 4.04E-02 | [ARID5B, MPC1, PIGA, TNFSF11] |
| GO:0005882 | intermediate filament | GO CC | 3.06E-15 | 5.12E-06 | [KRT10, KRT12, KRT20, KRT222, KRT23, KRT24, KRT25, KRT26, KRT27, KRT28, KRT39, KRT40, KRTAP1-1, KRTAP3-1, KRTAP3-3] |
| GO:0045111 | intermediate filament cytoskeleton | GO CC | 1.30E-13 | 5.12E-06 | [KRT10, KRT12, KRT20, KRT222, KRT23, KRT24, KRT25, KRT26, KRT27, KRT28, KRT39, KRT40, KRTAP1-1, KRTAP3-1, KRTAP3-3] |
| GO:0099513 | polymeric cytoskeletal fiber | GO CC | 1.06E-08 | 5.12E-06 | [DYNLT1, KRT10, KRT12, KRT20, KRT222, KRT23, KRT24, KRT25, KRT26, KRT27, KRT28, KRT39, KRT40, KRTAP1-1, KRTAP3-1, KRTAP3-3, MYO6] |
| GO:0099512 | supramolecular fiber | GO CC | 1.51E-07 | 5.12E-06 | [DYNLT1, KRT10, KRT12, KRT20, KRT222, KRT23, KRT24, KRT25, KRT26, KRT27, KRT28, KRT39, KRT40, KRTAP1-1, KRTAP3-1, KRTAP3-3, MYO6, RYR2] |
| GO:0003707 | steroid hormone receptor activity | GO MF | 7.01E-04 | 1.18E-02 | [ABHD2, NR1D1, RARA, THRA] |
| GO:0031490 | chromatin DNA binding | GO MF | 3.15E-03 | 1.18E-02 | [HMGN3, RARA, SMARCE1, THRA] |
| GO:0016298 | lipase activity | GO MF | 2.62E-02 | 1.61E-02 | [ABHD2, PLCB1, PLCZ1] |
| GO:0008081 | phosphoric diester hydrolase activity | GO MF | 1.43E-02 | 1.61E-02 | [PDE10A, PLCB1, PLCZ1] |
| GO:0043177 | organic acid binding | GO MF | 2.13E-03 | 2.34E-02 | [ACAN, GLRA2, HAPLN3, RARA, RYR2] |
| GO:0031406 | carboxylic acid binding | GO MF | 1.25E-02 | 2.34E-02 | [ACAN, GLRA2, HAPLN3, RARA] |
| KEGG:00562 | Inositol phosphate metabolism | KEGG | 1.84E-03 | 1.61E-02 | [PIK3C2G, PLCB1, PLCZ1, SYNJ2] |
| KEGG:04070 | Phosphatidylinositol signaling system | KEGG | 5.37E-03 | 1.61E-02 | [PIK3C2G, PLCB1, PLCZ1, SYNJ2] |
| KEGG:04919 | Thyroid hormone signaling pathway | KEGG | 8.45E-03 | 1.61E-02 | [MED24, PLCB1, PLCZ1, THRA] |
|  |  |  |  |  |  |
| **ID** | **Term** | **Ontology Source** | **Term PValue** | **Group PValue Corrected with Bonferroni (> 0.05)** | **Associated Genes Found** |
| GO:0031333 | negative regulation of protein complex assembly | GO BP | 2.69E-02 | 5.39E-02 | [CAPZA3, DNAJC15, THRA] |
| GO:0009743 | response to carbohydrate | GO BP | 2.47E-02 | 7.42E-02 | [HMGN3, NR1D1, RPS6KA2] |
| GO:0071322 | cellular response to carbohydrate stimulus | GO BP | 8.37E-03 | 7.42E-02 | [HMGN3, NR1D1, RPS6KA2] |
| GO:0042158 | lipoprotein biosynthetic process | GO BP | 2.13E-02 | 8.53E-02 | [PIGA, ZDHHC14, ZDHHC23] |
| GO:0006497 | protein lipidation | GO BP | 1.82E-02 | 8.53E-02 | [PIGA, ZDHHC14, ZDHHC23] |
| GO:0019933 | cAMP-mediated signaling | GO BP | 1.48E-02 | 8.79E-02 | [ADGRG6, DRD3, PDE10A] |
| GO:0019935 | cyclic-nucleotide-mediated signaling | GO BP | 1.76E-02 | 8.79E-02 | [ADGRG6, DRD3, PDE10A] |
| GO:0045095 | keratin filament | GO CC | 7.62E-03 | 5.33E-02 | [KRTAP1-1, KRTAP3-1, KRTAP3-3] |
| GO:0019897 | extrinsic component of plasma membrane | GO CC | 1.32E-02 | 7.90E-02 | [BMX, MFGE8, SNX9, SYTL3] |

Abbreviations. BP: Biological Process; MF: Molecular Function; CC: Cellular Component.
